# Influence of Nano-Methionine supplementation in drinking water on growth performance, lipid metabolism, and related gene expression in broiler chicken

**DOI:** 10.5455/javar.2022.i644

**Published:** 2022-12-31

**Authors:** Set A. El-Shobokshy, Magda I. Abo-Samaha, Eman M. Abd El-Maksoud, Shymaa A. Khatab, Asmaa F. Khafaga, Gemechu Wirtu

**Affiliations:** 1 Department of Nutrition and Veterinary Clinical Nutrition, Faculty of Veterinary Medicine, Alexandria University, Alexandria, Egypt; 2 Poultry Breeding and Production, Department of Animal Husbandry and Animal Wealth Development, Faculty of Veterinary Medicine, Alexandria University, Alexandria, Egypt; 3 Biochemistry Department, Faculty of Veterinary Medicine, Alexandria University, Alexandria, Egypt; 4 Genetics and Genetic Engineering, Department of Animal Husbandry and Animal Wealth Development, Faculty of Veterinary Medicine, Alexandria University, Alexandria, Egypt; 5 Pathology Department, Faculty of Veterinary Medicine, Alexandria University, Alexandria, Egypt; 6 Department of Biomedical Sciences, College of Veterinary Medicine, Tuskegee University, Tuskegee, AL, USA

**Keywords:** Broiler, Nano-Methionine, growth performance, lipid, gene expression, histopathology

## Abstract

**Objectives:**

The study aimed to determine how Nano-Methionine (Nano-Meth) affected growth, lipid metabolism, and relative gene expression for acetyl-CoA carboxylase (ACC), fatty acid synthase (FAS), growth hormone receptor (GHR), insulin-like growth factor receptor-1 (IGFR-1), myostatin (MSTN), and cholecystokinin (CCK) genes in broiler chickens.

**Materials and Methods:**

A total of 100 1-day-old broilers were randomly assigned into 2 groups: 1) the control group received drinking water without any supplements, and 2) the Nano-Meth group received 10 ml/l of 5% Nano-Meth starting from 1 day old until 35 days old (the end of the experiment).

**Results:**

Nano-Meth improved final body weight, weight gains, feed intake, and feed conversion ratio. Compared to the control group, Nano-Meth significantly lowered the serum levels of triglyceride, cholesterol, very low-density lipoprotein, and low-density lipoprotein in chickens. Nano-Meth significantly increased the serum levels of total protein, albumin, high-density lipoprotein, and glucose more than the control group. Nano-Meth lowered the mRNA gene expression of ACC, FAS, MSTN, and CCK but increased that of GHR and IGFR-1.

**Conclusions:**

We concluded that supplementation with Nano-Meth enhances growth performance and decreases lipid accumulation in broiler chickens.

## Introduction

Traditional poultry diets basically consist of corn and soybean meals, which are methionine-deficient [1]. Methionine (Meth) is a significant methyl group donor during protein metabolism. It is vital for feather growth and immune system function and is considered the first limiting amino acid in poultry [2]. Meth is essential for the development of the digestive tract and growth performance. Moreover, it is crucial for the development of feathers, muscle mass, antioxidant activity, and improving egg production in poultry [3–6].

Methionine requirements in feed vary according to chicken age and production status. The levels are 0.50%–0.52% during the starter, 0.38%–0.45% for the grower, and 0.32%–0.44% for the finisher phases of the production stage of broiler chickens [7,8]. DL-Methionine is the most commonly supplemented synthetic Meth product [9]. Other studies on broiler chicken used nano-iron plus methionine [10] or methionine-coated zinc nanoparticles [11]. When compared to organic zinc, supplementation with methionine-coated nano-zinc oxide improved broiler weight gain and feed intake (FI) significantly. Additionally, another study transformed DL-methionine into nano-particles and used it in broiler diets to examine its effects on growth, carcass traits, and intestinal morphology. They found that DL-methionine (7.5% and 10%) significantly increased the villus’ height, width, and absorptive surface area compared to the control [12].

Increases in abdominal and visceral fat mass and total body fat are common in growing chickens [13]. These deposits affect the juiciness, flavor, and taste of the meat. Fast-growing chickens deposit fat earlier and more quickly than slow-growing chickens [14]. However, selecting fast-growing broiler chickens makes the chickens more susceptible to stress, increasing the risk of metabolic disorders such as the fatty liver. As a critical amino acid, dietary Meth level is proven to affect such disorders in lipid metabolism in poultry [15,16]. Many reports have indicated that the addition of Meth affected abdominal fat deposition, blood lipid profiles, and levels of apolipoproteins [17,18], but a few studies addressed the hepatic lipid accumulation and the underlying mechanism [19], which revealed that birds given the low-methionine diet had an increased percentage of abdominal fat as compared to birds given an adequate Meth diet [20]. Met-supplementation reduced abdominal fat in treated broiler chickens [21] or white Pekin ducks [4,22].

The discovery of novel means of improving growth performance and stimulating muscle growth with low adiposity is essential. Till now, there are no reports about Nano-Methionine (Nano-Meth) and its effect on lipid metabolism in chicken; therefore, we designed this study to evaluate the effects of Nano-Meth supplementation on growth performance, lipid metabolism, and histopathological changes in the liver of broiler chicken. We also looked at the mRNA expression of some genes that affect growth [growth hormone receptors (GHR), insulin-like growth factor-1 (IGF-1), cholecystokinin (CCK), and myostatin (MSTN)] and lipid metabolism [acetyl-CoA carboxylase (ACC), and fatty acid synthase (FAS)].

## Material and Methods

### Ethical approval

All animal and experimental protocols were authorized by the Institutional Animal Care and Use Committee of the University of Alexandria (Permit #: 2022/013/126).

### Animal and feed

A 100 One-day-old Cobb-500 broiler chicks were randomly split into two groups: control and Nano-Meth. Each group had 50 chicks. The control group birds received a regular diet and drinking water. In contrast, birds in the Nano-Meth group were supplemented with Meth from day 1 until the end of the experiment (35 days). Nano-Meth was prepared at a concentration of 5%, and 10 ml of this was supplemented in a liter of drinking water, resulting in a final concentration of 0.05%. Nano-Meth was prepared according to Hojo et al. [23].

The chicks were raised on the floor in an environmentally controlled room. The temperature began at 32°C and then gradually decreased by 3.5°C per week until it reached 24°C. On the first day after arrival, there was a continuous 24-h lighting program, followed by a 23-h light:1-h dark schedule. Both groups were provided with a basal diet, shown in Table 1, per the recommendations of the National Research Council [7]. Formulated diets were analyzed for the major nutrients. Broiler chicks were fed on a starter ration during the first 2 weeks, then a grower ration during 3–4 weeks, and finally a finisher ration during 5^th^ week. The detailed composition and chemical analysis of the diets used for the starter, grower, and finisher periods are presented in Table 1.

**Table 1.** Ingredient composition and chemical analysis of the diets used in the experiment.

	Starter	Grower	Finisher
Ingredients			
Yellow corn (7.8% CP)	54.05	60.55	67.05
Soybean meal (44% CP)	32.75	29	24
Corn gluten (60% CP)	7.5	6	5
Vegetable oil^a^	1.8	1.1	1
MCP^b^	1.3	0.9	0.65
Limestone^c^	1.9	1.8	1.65
Lysine^d^	0.05	0.05	0.05
DL-Methionine^e^	0.1	0.05	0.05
Salt	0.25	0.25	0.25
Premix (mineral and vitamin)^f^	0.3	0.3	0.3
Total	100	100	100
Chemical analysis			
Moisture%	11.9	12.1	12
Crude protein%	23.12	21.07	18.7
Ether extract %	6.1	5.9	5.7
Ash%	6	5.9	6.1
ME kcal/kg diet	3,020	3,047	3,107

a Mixture of sunflower and soybean oils.

b Mono phosphate: contain 16% calcium and 21% phosphorus.

c Limestone contains 37% calcium and is locally produced.

d Lysine 87% produced by Archer Daniels Midland Company De Caur LL. Made in USA.

e DL-methionine produced by Evonik Co. Guaranteed analysis 99.5% DL-methionine.

f Each 3 kg contains: Vit A (12000000IU), Vit D (2000000IU), Vit E(10 gm), Vit K_3_ (2 gm), Vit B_1_ (1 gm), Vit B_2_ (5 gm), VitB_6_ (1.5 gm), Vit B_12_ (10 gm), Nicotinic acid (30 gm), Pantothenic acid (10 gm), Folic acid (1 gm), Biotin (50 mg), Choline chloride 50% (250 mg), Iron (30 gm), Copper (10 gm), Zinc (50 gm), Manganese (60 gm), Iodine (1 gm), Selenium (0.1 gm), Cobalt (0.1 gm) and carrier limestone up to 3 kg.

### Chemical analysis for the ration: dry matter and crude nutrients

The dry matter content of the ration sample was determined by oven-drying at 105°C for 8 h [24]. Ash content was characterized by incineration at 550°C overnight. Crude protein was determined using the Kjeldahl method according to Randhir and Pradhan [25], and ether extract was determined according to Hanson and Olly [26]. 

### Growth performance and carcass traits

Chicks were weighed individually at 1 day old (initial weight), then weekly till the end of the experiment (5 weeks). The differences between two successive weights (expressed in grams) were defined as weight gain, while the total gain (Tg) is the difference between the final and initial body weights (BWs). FI was calculated as the difference between the weight of offered feed and the remaining feed, then divided by the number of birds in each group per day and totaled to be per week, along with total FI. The feed conversion ratio (FCR) was calculated by dividing the amount of feed consumed (gm) during the week by the BW gain (gm) during the same week, as well as the average FCR. For carcass traits, nine broilers per group were weighed individually before slaughter (live weight). Following slaughter, the dressing percentage was calculated as carcass weight/live BW × 100. The organs such as the liver, gizzard, proventriculus, heart, thymus, and abdominal fat were removed from the carcass and weighed to determine the relative organ weight (%) = organ weight/live BW × 100. 

### Blood chemistry

Blood samples (nine from each group) were collected through brachial veni-puncture, and the sera were separated to estimate the serum glucose level [27]. Serum total cholesterol, triacylglycerol, high-density lipoprotein (HDL), and low-density lipoprotein (LDL) were measured according to Anwar et al. [28]. Serum total protein and albumin were analyzed using commercially available enzymatic spectrophotometric kits (Vitro Scient Kits, Cairo, Egypt).

### Gene expression

Liver and jejunum samples were taken from the chicken (three samples/group) and snap-frozen in liquid nitrogen and maintained at −80°C till subsequent use in RNA extraction. Quantitative real-time reverse transcription polymerase chain reaction (qRT-PCR) analysis of mRNA expression for growth-related genes (GHR, IGF1, and MSTN), fat metabolism genes (ACC), and FAS from the liver, CCK from the jejunum, and 18s (as a reference gene) was performed using the primers shown in Table 2 with designed primers using Primer 3 programs, (www.ncbi.nlm.nih.gov/tools/primerblast/primertool.cgi).

The total RNA was extracted from the samples using Easy-Red (Intron Biotechnology, Inc. cat. no. 17063). The quality of the extracted RNA was confirmed using Nanodrop (UV-Vis spectrophotometer Q 5000/USA). Isolated RNA from each group was used for cDNA synthesis with the SensiFAST^TM^ cDNA Synthesis Kit (Bioline, UK), according to the manufacturer’s instructions. The obtained cDNA was stored at −20°C until further use. The cDNA was checked by PCR using the housekeeping gene (18s). The product was checked on a 2% agarose gel and visualized by the gel documentation system (Biometra, Analytic Jena Company, Germany). The reaction (20 μl) consisted of 10-μl SYBR green with low Rox (Bioline, UK), 0.8 μl of each primer (F and R), 2 μl of cDNA, and 6.4 μl of RNase-free water. The program was conducted by initial heating at 95°C for 10 min followed by 40 cycles of 95°C for 15 sec and annealing temperatures specific to each primer (Table 1) for 1 min. The dissociation curve was generated at the end of the last cycle by collecting the fluorescence data at 60°C and taking measurements every 7 sec until the temperature reached 95°C to validate the specificity of the PCR amplicons.

**Table 2.** Primers sequences used in qRT-PCR.

Reference, acc. no.	Annealing temperature	Primer sequence (5'-3')	Gene
NM_205505	63	F:AATGGCAGCTTTGGAGGTGT R:TCTGTTTGGGTGGGAGGTG	ACC
J03860	63	F: CTATCGACACAGCCTGCTCCT R: CAGAATGTTGACCCCTCCTACC	FAS
El-Naggar et al. [29]	60	F: CACCTAAATCTGCACGCT R: CTTGTGGATGGCATGATCT	IGF-I
El-Naggar et al. [29]	60	F: AACACAGATACCCAACAGCC R: AGAAGTCAGTGTTTGTCAGGG	GHR
Dushyanth et al. [30]	60	F: GCAAAAGCTAGCAGTCTATG R: TCCGTCTTTTTCAGCGTTCT	MSTN
Song et al. [31]	60	F: CAGCAGAGCCTGACAGAACC R: AGAGAACCTCCCAGTGGAACC	CCK
Primer 3 XM_288030.1	60	F: CGAAAGCATTTGCCAAGAAT R: -GGCATGGTTTATGGTCGG	18s

### Histopathological study

Representative liver and jejunum samples from control and Nano-Meth-treated chicks were collected. The collected specimens were cleaned before being fixed for 24 h in a 10% neutral-buffered formalin solution. Then, specimens were embedded using Bancroft and Gamble’s paraffin-embedding procedure [32]. The preserved specimens were dehydrated in increasing concentrations of ethyl alcohol, cleaned in three changes of xylol, blocked in paraffin wax, and cut into 3–5 m thick pieces. Hematoxylin and eosin (H&E) was used to stain the sections. After that, a pathologist performed a blinded evaluation. Representative images were captured using a Leica DM500 microscope and digital camera (Leica EC3; Leica, Germany).

### Histomorphometric analysis of intestine

The birds were cut open after the blood was extracted, and the whole digestive tracts were separated. Further collections were made of the digestive tract’s middle segment. A digital camera attached to a bright field microscope was used to capture images from the digestive tissues of chicks (Nikon E200, Tokyo, Japan). After that, a computerized quantitative histomorphometric analysis of the photographs was performed. The following parameters were studied using light microscopic observations: (1) villus height (µ, from the tip to the base of villus); (2) villus width (µ, at the tip; and the crypt/villus junction); (3) crypt depth; (4) Villus height/crypt depth ratio; and (5) tunica muscularis thickness. The measurements were carried out using a computerized image analysis system (Image J software; Bethesda, MD) [33]. The mean value was calculated for 15 villus/samples (×10).

### Statistical analysis

Statistical analysis of growth performance parameters was performed by *t*-test using SAS software [34]. Data analysis of gene expression was done by comparative threshold cycle method 2−ΔΔct [35]. In this context, the fold change for each gene was normalized against the housekeeping gene (18s) and corresponding values of the control group. The overall significance level was set as *p* < 0.05. Statistical analysis of the gene expression data was performed using GraphPad Prism 6 software (GraphPrism Software, La Jolla, CA). 

## Results

### Growth performance

Data for BW, gain, FI, and FCR are shown in Table 3. BW did not differ between the two groups at day old, week 1, and week 2, while starting from week 3 till final BW (week 5), there was a significant difference with the Nano-Meth group weighting higher than the control group. Weight gain was significantly different in the first week, while it did not differ between groups in the second and third weeks. However, weight gain during the fourth and fifth weeks and the Tg were significantly greater in the Nano-Meth group than in the control group. The Nano-Meth group gained 250 gm more than the control group at the end of the experiment (5 weeks old). Nano-Meth significantly (*p* < 0.05) increased the FI during the whole experiment compared to the control group. Nano-Meth enhanced the FCR, with no significant difference between groups during the second and third weeks. However, a significant difference between the first, fourth, and fifth weeks and the average FCR was recorded.

### Carcass traits

Results for carcass traits are presented in Table 4. Dressing percentage, liver, gizzard, and heart did not differ between groups. Proventriculus and thymus were significantly increased in the Nano-Meth group when compared to the control group. However, abdominal fat decreased significantly in the Nano-Meth group compared to the control group. 

### Blood chemistry

Table 5 represents the statistical analysis data for blood chemistry. The triglyceride (TG), cholesterol, globulin, very low-density lipoprotein (VLDL), and LDL were significantly lower in the serum of chickens given Nano-Meth than those non-supplemented chickens (*p < *0.05). Moreover, adding Nano-Meth significantly increases the serum total protein, albumin, HDL, and glucose level.

**Table 3.** Effect of Nano-Methionine on BW, weight gain, FI, and FCR.

Variable	Group		*t* value	Pr (>|*t*|)
	Control	Nano-Methionine		
Day old	49.66 ± 0.53	49.01 ± 0.29	1.06	NS
Week 1	144.63 ± 3.74	153.71 ± 3.48	−1.78	NS
Week 2	428.59 ± 11.52	443.02 ± 10.33	−0.93	NS
Week 3	924.74 ± 20.17	987.82 ± 17.23	−2.38	0.0194
Week 4	1,476.28 ± 25.96	1,630.50 ± 23.26	−4.42	<0.0001
Week 5	2,055.46 ± 38.87	2,304.61 ± 30.90	−5.02	<0.0001
G1 (day old–W1)	94.97 ± 3.25	104.70 ± 3.20	−2.13	0.0354
G2 (W1–W2)	283.96 ± 10.12	289.31 ± 10.03	−0.38	NS
G3 (W2–W3)	496.15 ± 19.03	544.80 ± 18.44	−1.84	NS
G4 (W3–W4)	551.54 ± 7.48	642.68 ± 7.48	−8.61	<0.0001
G5 (W4–W5)	579.17 ± 15.86	674.12 ± 11.15	−4.9	<0.0001
Tg (day old–W5)	2,005.80 ± 38.34	2,255.60 ± 30.62	−5.09	<0.0001
FI (day old–W1)	138.90 ± 0.35	137.68 ± 0.36	2.44	0.0167
FI2 (W1–W2)	390.90 ± 0.24	395.00 ± 0.39	−8.99	<0.0001
FI3 (W2–W3)	685.00 ± 0.42	730.00 ± 0.16	−100.42	<0.0001
FI4 (W3–W4)	933.10 ± 0.28	998.90 ± 0.25	−175.95	<0.0001
FI5 (W4–W5)	1,221.90 ± 0.24	1,238.70 ± 0.43	−34.08	<0.0001
TFI (day old–W5)	3,368.90 ± 0.64	3,500.28 ± 0.43	−169.63	<0.0001
FCR1 (day old–W1)	1.59 ± 0.09	1.37 ± 0.04	2.37	0.0197
FCR2 (W1–W2)	1.59 ± 0.15	1.48 ± 0.08	0.63	NS
FCR3 (W2–W3)	1.51 ± 0.08	1.47 ± 0.09	0.34	NS
FCR4 (W3–W4)	1.70 ± 0.02	1.56 ± 0.02	5.32	<0.0001
FCR 5 (W4–W5)	2.19 ± 0.06	1.86 ± 0.03	4.83	<0.0001
FCR (average)	1.71 ± 0.03	1.57 ± 0.02	3.77	0.0003

Data are presented as mean ± standard error with (*p* < 0.05).

NS: Non-significant; G: gain; FI: feed intake; Tg: Total gain, TFI: total feed intake, FCR: feed conversion ratio.

**Table 4.** Effect of Nano-Methionine on carcass traits.

Variable	Group		*t* value	Pr(>|*t*|)
	Control	Nano-Methionine		
Dressing %	69.94 ± 0.15	70.90 ± 1.08	−0.88	NS
Liver	2.33 ± 0.03	2.26 ± 0.05	1.02	NS
Gizzard	1.36 ± 0.01	1.36 ± 0.03	−0.07	NS
Proventriculus	0.34 ± 0.01	0.40 ± 0.01	−4.88	0.0002
Heart	0.43 ± 0.01	0.40 ± 0.01	1.97	NS
Abdominal fat	1.28 ± 0.04	0.97 ± 0.02	7.94	<0.0001
Thymus	0.30 ± 0.02	0.39 ± 0.01	−4.44	0.0004

The data presented as mean ± standard error with (*p* < 0.05).

NS: Non-significant.

**Table 5.** Effect of Nano-Methionine on selected blood chemistry parameters.

Variable	Group		*t* value	Pr(>|*t*|)
	Control	Nano-Methionine		
Total protein (gm/dl)	5.36 ± 0.01	5.43 ± 0.01	−4.54	0.0003
Albumin (gm/dl)	3.50 ± 0.09	3.96 ± 0.01	−5.34	<0.0001
Globulin (gm/dl)	1.86 ± 0.08	1.47 ± 0.03	4.48	0.0004
Alb/Glob	1.93 ± 0.14	2.69 ± 0.05	−4.95	0.0001
Glucose (mg/dl)	111.37 ± 0.28	129.88 ± 0.69	−24.94	<0.0001
Cholesterol (mg/dl)	198.72 ± 1.68	186.93 ± 0.66	6.55	<0.0001
Triglyceride (mg/dl)	200.18 ± 0.59	187.25 ± 0.48	16.99	<0.0001
HDL (mg/dl)	50.57 ± 0.26	58.50 ± 0.30	−19.73	<0.0001
LDL (mg/dl)	108.12 ± 1.41	90.98 ± 0.83	10.46	<0.0001
HDL/LDL ratio	0.47 ± 0.01	0.65 ± 0.01	−17.52	<0.0001
VLDL (mg/dl)	40.04 ± 0.12	37.45 ± 0.10	16.99	<0.0001
Cholesterol/HDL	3.93 ± 0.02	3.19 ± 0.02	25.35	<0.0001

Data presented as mean ± SE.

HDL: high-density lipoprotein, LDL: low-density lipoprotein, VLDL: very low-density lipoprotein.

### Gene expression

**Figure 1.**
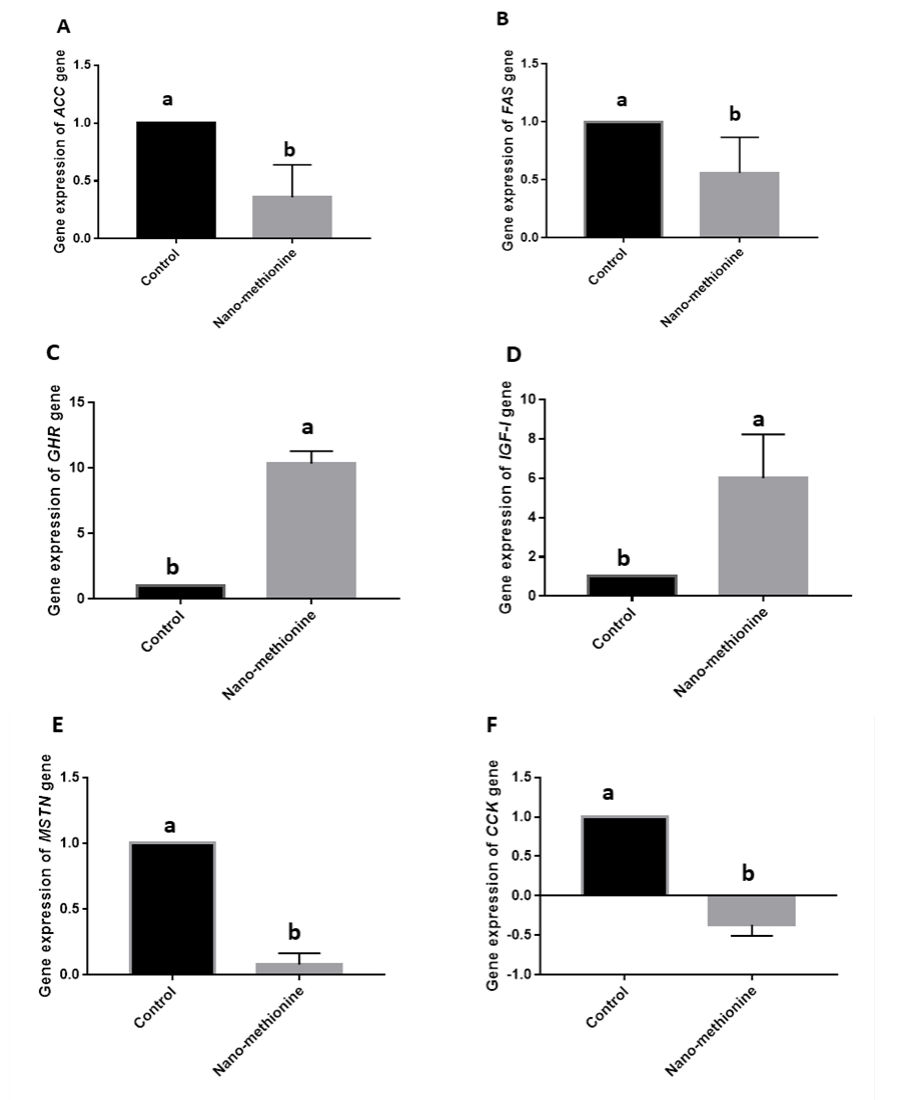
Relative expression of selected genes with effects on fat metabolism in the liver: (A) ACC, (B) FAS; affecting growth: (C) GHR, (D) IGF-1, (E) MSTN, and (F) CCK in response to Nano-Methionine supplementation in broiler chicken. All values are expressed as mean ± SE. Acetyl-coA carboxylase (ACC), fatty acid synthase (FAS), growth hormone receptor (GHR), insulin-like growth factor receptor -1 (IGFR-1), myostatin (MSTN), cholecystokinin (CCK).

The mRNA expressions of the fat metabolism genes, ACC, and FAS showed a significant down-regulation to 0.36 ± 0.28 and 0.56 ± 0.30 fold, respectively (Fig. 1A and B) in the Nano-Meth supplemented broilers. The supplementation with Nano-Meth caused a significant increase in the expression of GHR and insulin-like growth factor receptor-1, to 10.3 + 0.95 and 6 + 2.3-fold, respectively (Fig. 1C and D). However, it caused a significant decrease in the expression of MSTN (Fig. 1E) when compared to the control group. However, our results showed a significant down-regulation of the CCK gene (−0.36 + 0.14-fold in the Nano-Meth group compared to the control). 

### Histopathologic findings

#### Liver

**Figure 2.**
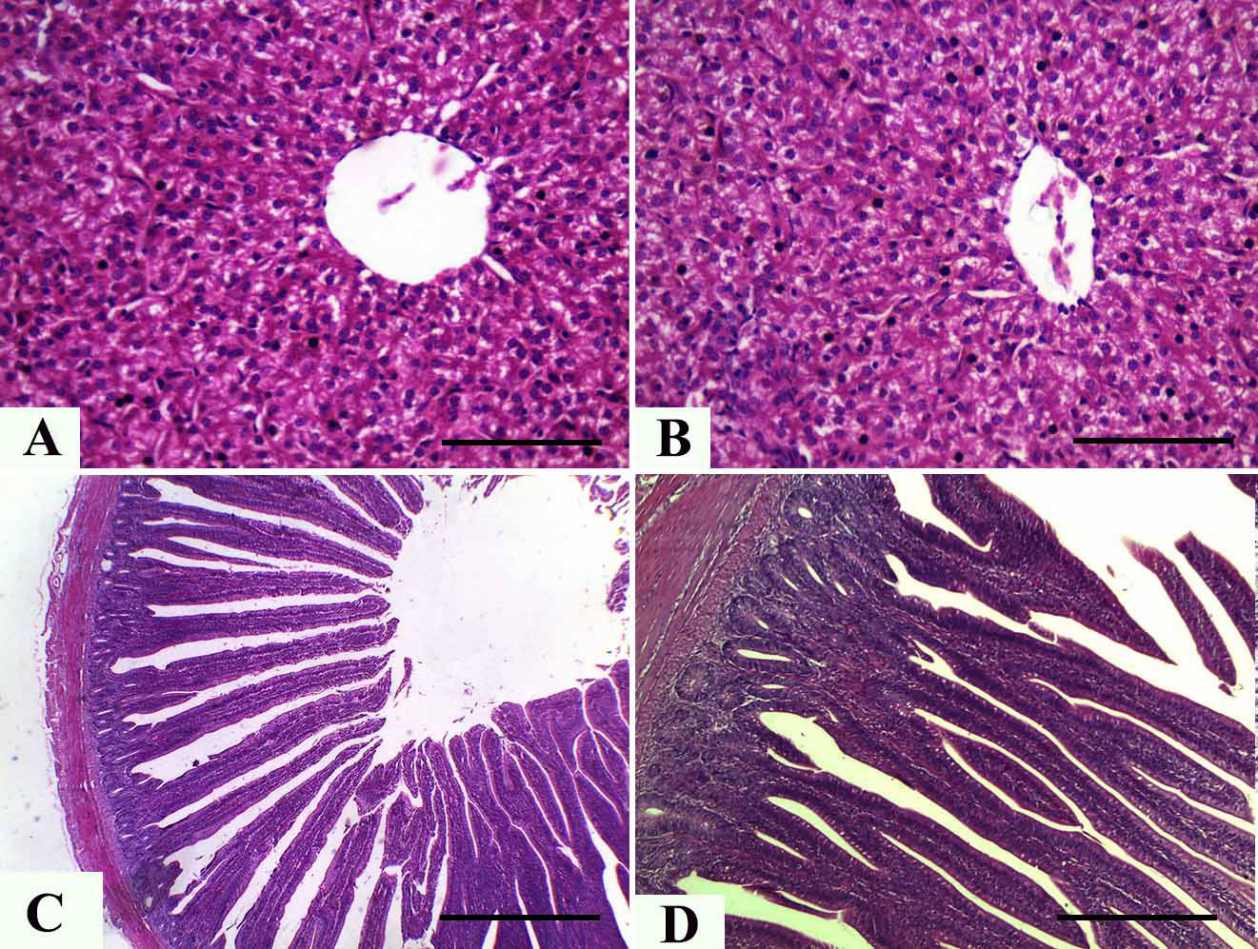
Representative photomicrographs (H&E staining) for hepatic and intestinal tissues from control (A and C) and Nano-Methionine-treated (B and D) chicken showing: (A and B) normal histologic architecture of liver parenchyma with polygonal hepatocytes contain round shape nucleus and adequate amount of cytoplasm as well as normal structure of central vein and sinusoids. (C and D) Normal histomorphologic structure of intestinal villi and associated crypt as well as the internal and external muscular layers, the submucosal tissues showed normal histologic limits with proper arrangement of goblet cells and columnar epithelium, without evidence for degenerative and/or inflammatory changes. *Bar* = 50 µm for A, B, and D. *Bar* = 200 µm for C.

The results from the histopathologic examination of hepatic tissues from both control and Nano-Meth-treated chickens showed normal histologic architecture of liver parenchyma; the hepatocytes appeared with a polygonal shape, contained a round nucleus, and contained an adequate amount of cytoplasm. Moreover, the portal triads, central and portal veins, hepatic artery, and sinusoids showed normal structures (Fig. 2A and B). 

#### Intestine

As shown in Figure 2, the histologic structure of the chicken intestinal wall was examined for its four layers (tunica serosa, internal and external tunica muscularis, submucosa, and mucosa). The intestinal mucosa and submucosa of the control (Fig. 2C) and Nano-Meth-treated chicken (Fig. 2D) showed the normal histomorphologic structure of intestinal villi and associated crypt. Similarly, the internal and external muscular layers did not show any deviation from normal architecture and morphology. Additionally, the submucosal tissues showed normal histologic limits with proper arrangement of goblet cells and columnar epithelium without evidence for degenerative and/or inflammatory changes.

**Table 6.** Histomorphometric analysis of intestines from chickens supplemented with Nano-Methionine.

Measurement (µm)	Control	Nano-Methionine
Villus height	597.3 ± 16.3	629 ± 3.6056
Villus width at the tip	53.3 ± 7.3	60 ± 1.7321
Villus width at the crypt/villus junction	54.0 ± 5.0	52.00 ± 5.2915
Crypt depth	71.3 ± 1.8	79.00 ± 3.6056
Villus height/crypt depth ratio	8.4 ± 0.1	7.993 ± 0.379
Tunica muscularis thickness	224.7 ± 3.2	230.3 ± 5.4874

Data represent mean ± SE.

The histomorphometric analysis of the intestinal tissues (Table 6) showed that the administration of Nano-Meth didn’t induce significant (*p* < 0.05) changes in the studied and calculated parameters (including villus height, villus width at the tip, the crypt/villus junction, crypt depth, the villus height/crypt depth ratio, and tunica muscularis thickness) as compared to the control group.

## Discussion

From our results, Nano-Meth supplementation in drinking water leads to improved BW, weight gain, FI, and FCR. Our results are consistent with others [36,37], who reported that broiler chicks given an L-met-supplemented ration showed better average daily gain and FCR. The explanation for this improvement in growth performance may be a return to the higher gene expression levels of GHR and IGF-1, reduced gene expression of MSTN, and downregulation of CCK. Another study found that the mechanism of action of Meth on growth is that improved use of Meth may improve intestinal growth, which results in better growth performance [36]. Another study showed that the Meth-free ration resulted in a significant decrease in average daily FI and feed efficiency in comparison to the Meth-sufficient group. Meth deficiency also resulted in a significantly reduced final BW and weight gain. However, the Meth-excessive ration did not affect growth performance compared to the Meth-sufficient group [38]. Additionally, Li et al. [39] indicated that feeding rations with low or excessive Meth levels in chickens resulted in negative impacts on growth. These negative impacts may be attributed to the Meth source, which was DL-Meth in their study.

In this study, Meth did not affect the dressing percentage, the heart, gizzard, or liver relative weight (*p* > 0.05) compared to the control. Similarly, Peng et al. [38], indicated that liver weight was the same in broilers fed a Meth-excessive and Meth-sufficient diet, while feeding the Meth-deficient diet decreased liver weight.

Our results showed that abdominal fat decreased with Nano-Meth supplementation. Similarly, Andi [21] found that 0.2% Meth in the starter ration reduced abdominal fat (*p* ≤ 0.05) when compared to control (1.58% *vs.* 2.5%, respectively) in broilers at 42 days old. Xie et al. [4] and Wang et al. [22] indicated significant reductions in abdominal fat percentage in White Pekin ducks from 21 to 48 days of age and Yangzhou geese from 28 to 70 days of age due to higher dietary Meth. These outcomes may be returned to the stimulatory action of Meth on the expression of genes coding for growth-related hormones and myogenic regulatory factors involved in muscle development [40]. The lower expression of lipogenic genes (ACC and FAS) and the reduction in serum TG and lipoprotein levels in this study, further support the effects of Meth on reducing abdominal fat content in broiler chicken.

We observed that supplementation with Nano-Meth increased total serum protein, albumin, and glucose levels more than those of the control group. Similarly, Ospina-Rojas et al. [41] and Jariyahatthakij et al. [42] showed that supplementing synthetic Meth in a low-crude protein (CP) ration increased the plasma total protein and albumin levels in broiler chickens. In contrast to the findings in our study, others reported that blood metabolites (e.g., Total Protein, Alb, and globulin) did not change throughout the experiment regardless of the sources [43] or levels of methionine [44].

The increased glucose level in the Nano-Meth-supplemented group in our study is most likely due to GH activation of hormone-sensitive lipase (HSL) in visceral adipose tissue, which stimulates free fatty acid (FFA) flow from adipose tissue to circulation. Increased circulatory FFA induces resistance to insulin through inhibition of insulin receptor substrate-1 activity and subsequent failure of PI3K activation in the skeletal muscle and liver. As a result, increased FFA uptake in the liver stimulates hepatic fatty acid oxidation and acetyl-CoA accumulation [45]. Acetyl-CoA stimulates pyruvate carboxylase and phosphoenolpyruvate carboxykinase (the two key enzymes for gluconeogenesis) and releases glucose from the liver and kidney into circulation, thus increasing blood glucose levels [46].

Our study showed that triglyceride (TG), cholesterol, VLDL, and VLDL in the serum of Nano-Meth-fed chickens were significantly reduced compared to the control group. Similarly, El-Wahab et al. [47] reported that broilers fed high dietary lysine, methionine, and L-carnitine showed lower levels of serum TG, cholesterol, and lipids when compared to the control group. Additionally, Kalbande et al. [18] indicated that high dietary Meth levels decreased the serum triglyceride concentration, although the serum cholesterol level was unaffected. In contrast, another study showed that dietary methionine levels did not affect serum triglyceride and total cholesterol levels [48]. The liver produces triglycerides that are transported into the blood as lipoproteins and then released under the effect of lipoprotein lipase (LPL) to be stored in adipocytes or catabolized in tissues [49]. Methionine supplementation lowers serum TG levels by inhibiting the hepatic secretion of TG-rich lipoprotein [50] and promoting LPL activity [49].

The lower expression level of FAS in the Nano-Meth group (Fig. 1 B) in this study may be related to increased LPL expression that has been reported to be associated with decreased FAS expression [51]. We also observed that Nano-Meth significantly decreased the gene expression level of ACC (Fig. 1A) compared to the control group. Similarly, Jariyahatthakij et al. [42] also found that supplementation of Meth to low-CP ration to broiler chickens tends to reduce ACC gene expression and significantly reduce the content of abdominal fat compared to the control group. Takahashi and Akiba [52] also reported that L-methionine exerts a fat-lowering effect by decreasing FAS activity (lipogenesis) and stimulating HSL activity (lipolysis). This explains the lower abdominal fat in the Nano-Meth group than in the control group in this study.

The present findings suggest that supplementation with Nano-Meth caused a significantly greater expression of the GHR and IGF1 genes (Fig. 1C and D) than the control group. Del Vesco et al. [53] indicated a similar finding that IGF-1 and GHR mRNA gene expression in the liver of broilers fed methionine-supplemented rations was higher than those of broilers fed a control diet. Meth has a major role in improving animal performance by stimulating the synthesis and production of growth factors in all cell types. IGF-1 is synthesized in the liver and secreted into the blood under the control of GH and is responsible for various physiological, and metabolic functions of cells and the metabolism in chickens [54]. IGF-1 and GHR are strong growth regulators and have anabolic effects on protein and carbohydrate metabolism [55]. IGF-1 is a vital regulator for the development of muscle as it amplifies cell protein content in broiler myofiber [56] and stimulates broiler myoblast proliferation. Moreover, IGF-1 stimulates embryonic skeletal muscle growth, and skeletal myogenesis, and consequently improves chicken meat production [57].

From our results, the lower expression level of the MSTN gene in the Nano-Meth group as compared to the control group (Fig. 1E) is associated with improved growth performance. The MSTN gene, which is one of the transforming growth factor β superfamilies, is a potent negative regulator for muscle development and differentiation [58]. Thus, the high expression of MSTN decreases muscle growth through the down-regulation of myogenic differentiation factor and myogenic factor expression levels [59]. Wen et al. [60] showed that dietary methionine supplementation for broilers enhanced breast muscle development, which resulted from the downregulation of MSTN gene expression. Interestingly, El-Saway et al. [61] indicated that methyl methionine sulfonium chloride supplementation improved growth performance and increased muscle mass via upregulation of IGF-1 mRNA expression and downregulation of MSTN mRNA expression.

CCK is an anorexigenic gut peptide in poultry. It is mainly released by intestinal epithelial cells, has a strong inhibitory effect on appetite, and induces intestinal motility [62]. CCKs are highly distributed in the gastrointestinal tract and are necessary for regulating FI in broilers and layers [63]. The use of exogenous CCK has also been reported both peripherally [64] and centrally [65] to reduce chicken FI. In this study, CCK downregulated significantly in the intestine of Nano-Meth broilers (Fig. 1F) compared to the control group. This may partly be the reason for the improvement in BW and weight gain of the Nano-Meth broilers due to increased FI [66]. Similarly, another study [67] showed that Meth did not trigger the release of CCK in the duodenum of pigs.

## Conclusion

Our data indicate that Nano-Meth is very effective in improving growth performance and decreasing abdominal fat content in broiler chickens through upregulation of GHR and IGF1, which enhance growth and muscle mass, and downregulation of regulatory enzymes for lipogenesis (FAS and ACC). Nano-Meth supplementation also lowered the serum lipid profile. Therefore, we strongly recommend the supplementation of Nano-Meth in broiler diets.

## List of abbreviations

ACC, acetyl-coA carboxylase; BW, body weight; CCK, Cholecystokinin; CP, Crude protein; FAS, fatty acid synthase; FCR, feed conversion ratio; FFA, free fatty acid; FI, feed intake; GHR, growth hormone receptor; HDL, high-density lipoprotein; IGFR-1, insulin-like growth factor receptor-1; LDL, low-density lipoprotein; Meth, methionine; MSTN, Myostatin; TG, triglyceride; VLDL, very low-density lipoprotein.
